# Missed opportunities for vaccination in Peru 2010–2020: A study of socioeconomic inequalities

**DOI:** 10.1016/j.lana.2022.100321

**Published:** 2022-07-18

**Authors:** Jose Matta-Chuquisapon, Camila Gianella, Gabriel Carrasco-Escobar

**Affiliations:** a Health Innovation Laboratory, Institute of Tropical Medicine “Alexander von Humboldt”, Universidad Peruana Cayetano Heredia, Lima, Peru; b Pontificia Universidad Católica del Perú, Peru; c School of Public Health and Administration, Universidad Peruana Cayetano Heredia, Lima, Peru; d Herbert Wertheim School of Public Health and Human Longevity Science, University of California San Diego, La Jolla, CA, USA

**Keywords:** Vaccines, Immunization, Immunization programs, Demographic and health survey, Healthcare disparities, Socioeconomic factors

## Abstract

**Background:**

Missed Opportunities for Vaccination (MOV) represent a major risk in the re-emergence of immunopreventable diseases. However, in the region, there are few published studies on MOVs using national databases such as demographic and health surveys (DHS). This study aims to describe the frequency and trends of MOVs for the first dose of vaccines against the leading causes of infant morbidity and mortality, their complete vaccination coverage, and trends in socioeconomic inequalities at the national and departmental levels for an 11-years period.

**Methods:**

Using DHS data from an 11-year period (2010–2020), we calculated frequencies and trends in MOVs of vaccines for the leading causes of child morbidity and mortality, estimated inequalities in MOVs using the Slope Inequality Index (SII) and conducted a spatial autocorrelation test to identify clusters of higher or lower inequality in MOVs at the national level.

**Findings:**

We found that, at the national level, greater inequality was concentrated in the wealthiest categories of each socioeconomic variable. We identified that departments with higher poverty rates concentrated higher levels of inequality in the MOVs in the lowest strata of the socioeconomic variables. In addition, we found that some departments with similar geographic and socioeconomic characteristics had spatially correlated levels of inequality on MOVs.

**Interpretation:**

These findings can help to identify the heterogeneity that exists in the distribution of MOVs among departments and socioeconomic strata, which would help to prioritize specific areas and subpopulations for national immunization strategies.

**Funding:**

No additional funding source was required for this study.

**Research in context**

**Evidence before this study**

Missed Opportunities for Vaccination (MOV) represent a major risk in the re-emergence of immunopreventable diseases that have already been eradicated or controlled in most countries. However, in the region there are few published studies on missed opportunities for vaccination using national databases such as demographic and health surveys (DHS), and specifically, there are no known published studies in Peru. A literature search was conducted in PubMed on October 27, 2021 with the following search strategy ((((("missed opportunities for vaccination"[Text Word]) OR ("missed opportunities"[tiab])) OR ("mov"[tiab])) AND (((("children 0 5 years"[tiab]) OR ("under 5 age"[tiab])) OR ("children"[tiab])) OR ("child"[MeSH Terms]))) AND ((("inequalities"[tiab]) OR ("socioeconomic"[tiab])) OR ("socioeconomic inequality"[tiab]))) AND (((("America"[tiab]) OR ("Americas"[tiab])) OR ("Sudamerica"[tiab])) OR ("Peru"[tiab])). No publication has been found that includes missed opportunities for vaccination in Peru or South America using nationally representative data, such as DHS.

**Added value of this study**

This is the first study published in Peru that uses 11-year DHS to describe the frequency and evolution of MOVs for five vaccines against the leading causes of mortality in children under five years old. Moreover, we estimate inequalities in MOV based on socioeconomic variables (Maternal education and Wealth index) at the national and departmental levels. We found that by 2010, the national levels of MOVs reached levels ranging from 45% (pentavalent vaccine) to 78% (rotavirus vaccine). At the national level, we found a greater concentration of inequities in MOVs in the higher socioeconomic strata. However, we identified that the departments with higher poverty rates concentrated higher levels of inequality on MOVs in the lowest strata of the socioeconomic variables. In addition, we found that some departments with similar geographic and socioeconomic characteristics had spatially correlated levels of inequalities on MOVs.

**Implication of all the available evidence**

These findings can help to identify the heterogeneity that exists in the distribution of MOVs among departments and socioeconomic strata, which would help to prioritize specific areas and subpopulations for national immunization strategies.

Alt-text: Unlabelled box

## Introduction

Vaccination is one of the most important achievements in public health responsible for reducing more than 90% of morbidity rates since its introduction, benefiting both children and adults.^1^^,^^2^ In 2010, the World Health Organization (WHO) published the Global Vaccine Action Plan (GVAP) 2011–2020, which aimed to reduce child mortality and meet immunization coverage targets in all countries, departments, and communities.^3^ Despite the global effort to meet GVAP aims, by the end of 2019, approximately 20% of children still lacked access to all WHO-recommended vaccination (pentavalent, pneumococcal, influenza, and rotavirus vaccines).^4^ The GVAP results showed that although vaccination coverage has increased (not at the expected levels), the benefits of immunization continue to be inequitably shared both between and within countries. This inequality has led to outbreaks of some already controlled diseases such as polio and measles.^4^ As of 2021, a new WHO plan called “Immunization agenda 2030” is being implemented. One of its aims is to close the gaps in vaccination coverage of preventable diseases and ensure that everyone has access to vaccines.^5^

For the study of the lack of vaccination, WHO recommends the health facility-based approach, as it allows to identify with greater precision the drivers to not provide vaccination to a child at the heath facility even though it is not contraindicated. This condition is known as missed opportunities for vaccination (MOV).^6^ WHO defines MOVs, for study purposes, as the non-vaccination status of a child under five years old who is eligible for vaccination and has access to health services.^7^ This condition is influenced by several factors, which can be grouped into those related to the health worker, the caregiver, and the health system.^6^ In Latin America, several unpublished studies (PAHO abstracts and national immunization program data) focusing on MOVs have identified that the main factors of MOVs are misinformation about contraindications in the child, attitude of health personnel that generates distrust and logistical problems.^8^

Both the strategic objective 3 of the GVAP and the immunization agenda 2030 target to eliminate inequalities in the vaccination of preventable diseases and MOVs.^8^ It is well known that socioeconomic inequalities have a high impact on low vaccination coverage and MOVs, with maternal education, income, and place of residence as some of the main factors.^9^ There are several methodologies focused on the study of these socioeconomic inequalities that affect health, such as the measures based on the ranking of the socioeconomic variable.^10^ In this group, the slope index of inequality (SII) measures the differences in the rate of a health event between the highest and lowest level of a socioeconomic variable.^11^ This index has the advantage of describing inequality of the whole population, takes the size of the population of each group for the calculation, contemplates a socioeconomic dimension in its analysis, and is sensitive to spatiotemporal changes when conducting clusters analyses.^10^^,^^12^ A rich source of demographic, socioeconomic, and health data to establish the relationship or impact of socioeconomic inequalities in health is the Demographic and Health Surveys (DHS).^13^ They provide the required data for this type of inequality measurement, although only a few countries carry them out frequently. Peru is one of the countries that has been conducting these surveys annually since 1996 and has incorporated them as a strategy to periodically measure the outcome of its policies.

In Peru's case, according to its latest technical norm on immunizations published by the Ministry of Health in 2018, it has 17 vaccines nationwide, which are distributed free of charge.^14^ It defines the target population as children under five years old, women of reproductive age, pregnant women and older adults.^14^ Among the 17 vaccines available, there are those which are obligatory, such as pentavalent, pneumococcal, rotavirus, influenza, polio, varicella, Influenza, MMR triple viral, and Hepatitis A vaccine.^14^ The compliance with this vaccination scheme and the application of mandatory vaccines for the target population is reinforced by strategic programs provided by the state. One of the most important programs is CRED, managed by the Peruvian Ministry of Health (MoH), and the social program JUNTOS, managed by the Ministry of Development and Social Inclusion. However, vaccination rates for the most critical vaccines in children under five years old are still lower than expected, and vaccination coverage varies between different subnational areas in the country.^15^ In this context, the present study uses data from the DHS to describe the frequency and trends of missed opportunities for vaccination of the first dose of vaccines against the main causes of child morbidity and mortality (pentavalent, rotavirus, influenza, and pneumococcal), their complete vaccination coverage, and estimate the SII using two socio-economic metrics at the national and departmental levels for an 11-year period.

## Methods

### Study area

Peru is an upper-middle income country located in South America (9°11′23.9″S, 75°00′54.6″W). It is a multi-ethnic country, with population mostly concentrated in urban areas, especially in Lima, the city with the highest population growth due to massive migrations since the 20th century. It is divided in 24 regions, called departments, each one with authorities responsible for the development and administration of the region. In regards to health, it has a national vaccination schedule that starts at birth and ends over 65 year old population. These vaccinations are applied for free at public health facilities. The first doses of pneumococcal, influenza, rotavirus and pentavalent vaccines (included in this study) are administered in the first six months of life, completing the vaccination schedule, including boosters, before five years of age.^14^ It is also important to mention that, despite of having recommended vaccination schedules, these are flexible and can be administered even if the child is older than what is established in the national vaccination schedule, although with certain time limits.^14^ The vaccination schedule by age is detailed in Supplementary Table 1.

### Study design

A retrospective data analysis of the Demographic and Health Survey (DHS) conducted by the National Institute of Statistics and Informatics of Peru.^16^ Annual surveys from 11 years (2010-2020) were analyzed to determine the frequency and evolution of MOVs for each vaccine (pentavalent, rotavirus, influenza, and pneumococcal) and estimate the inequality in MOVs based on different socio-economic variables. We used maternal education and wealth index as socioeconomic variables to estimate the Slope Index of Inequality (SII), due to its impact on health described elsewhere.^17^, ^18^, ^19^ The SII was calculated at national and departmental levels for the 11-year period. Besides, a sub-analysis of SII was conducted stratifying the population by residence type, access to social programs (JUNTOS program), and place of attention to the growth and development control program (CRED by its acronym in Spanish).

### Data source

DHS aims to update information on demographic dynamics, the health of mothers and children under five years old, and to obtain information on transmissible and non-transmissible diseases present in the general population. This survey is conducted annually and collects health, housing, nutritional, immunization, and socioeconomic data that can be extrapolated to the national level. The level of inference provided by this survey is at national, national-rural, national-urban and department levels.^16^ For this purpose, a stratified, two-stage, probability sampling design was used. The primary sampling units (PSUs) consisted of clusters of households and the secondary sampling unit (SSU) is composed of individual households that belong to the PSU clusters. These PSUs were different in urban and rural areas. In the case of urban areas, the block or group of blocks that includes 120 households is considered a PSU. For rural areas, there is no distribution of households by blocks, but by population centres or communities. Communities of at least 500 people up to a maximum of 2000 were considered as a PSU. All the details of the sampling design can be found in the technical sheet of the survey.^16^ It is important to mention that, since 2015, the sampling method was changed to a balanced sampling (cube method) which allows maintaining the proportions of the original population in the sample on some equilibrium variables.^16^ This sampling method has been used due to the high variability detected in the estimation of the basic estimators in previous surveys.^16^

### Definition of variables

For this study, we defined “MOV” as non-vaccination status in children under five years who had access to a health service. For this, we defined “access to health services” as the presence of at least one of the following two indicators: mother had health insurance or belonging to the CRED program provided by the MoH of Peru. The CRED program is a national strategy to ensure the optimal development of children by monitoring nutrition, immunization, growth and development from birth to five years old.^20^ Enrollment in this program is free and given nationwide at all levels of care. For the calculation of inequality metrics, we use socioeconomic variables such as maternal education (ME), which is based on the maximum level achieved by the mother of a child under five years old, and the wealth index (WI), which is a composite measure of a household's cumulative standard of living, calculated from a principal components analysis of housing characteristics, basic sanitation, ownership of consumer durables, type of cooking fuel, and number of persons per room.^21^^,^^22^ The “residence type” is defined as the place where the household is located (rural or urban), and the “place of attention of CRED”, defined by whether it is provided in a public (government) or private facility. JUNTOS is a social program created in 2005 that provides conditional cash transfers to ensure maternal and child health and education for Peru's poorest households, to remove them from this situation.

### Spatiotemporal description of vaccination and socioeconomics

To calculate the relative and absolute frequencies with their 95% confidence interval for each variable, the clusters, the strata, and the weighting factor were considered for all the variables used in the analysis. We describe the main health and socio-demographic characteristics related to vaccination for the 11-year period at national and departmental levels. We describe the evolution of vaccination coverage for each vaccine included and describe the MOVs for the first dose of each vaccine, that is, the missed opportunity to start the vaccination schedule. Moreover, we evaluate the national and departmental evolution of socio-demographic characteristics such as residence type, ME WI, place of attention of CRED, and access to social programs such as JUNTOS.

### Estimation of inequalities in vaccination by SII

The inequality metric that we used in this study was SII. The SII represents the coefficient of a linear relationship between the frequency or rate of a health problem in each socioeconomic category and the hierarchical ranking of each socioeconomic category on the social scale. The SII was calculated using a Poisson regression with robust estimations, with log link function between the rate of MOVs, and the hierarchical ranking of two socioeconomic variables (Maternal Education and Wealth index). SII was interpreted as the absolute change in the health event (in this case, rate of MOVs) when moving from the lowest level of the socioeconomic variable (rank=0) to the highest level (rank=1).^10^ The calculation was made for the data at the national level and, additionally, the index was calculated for each department.

#### Spatial autocorrelation analysis

The spatial autocorrelation of the SII (the estimates in each department are associated with the values obtained in neighbouring departments) was evaluated at the national level using *Moran's I test*.^23^ A first-order queen contiguity-based weighted neighbourhoods (departments with at least one contiguous boundary vertex) was used for the calculation of the test. The analysis was conducted for each year of the period 2010–2020.

#### Stratified analysis

The stratification of the inequity analysis using the SII was done to obtain a more comprehensive analysis of inequities in MOVs. We stratified the analysis by residence type, place of attention of CRED, and access to social programs focused on maternal and child health, such as the JUNTOS program. Data processing and consolidation, descriptive analysis, calculation of indicators and visualization of results were performed in R software v.4.0.1.^24^

### Role of the funding source

None.

## Results

### Spatiotemporal trends of socioeconomics variables, vaccine coverage, and MOVs

The distribution of each child's residence type has been stable during the years 2010 to 2014 with an average of 65% for urban areas, until 2015, when an increase above 70% was observed for housing in urban areas, maintaining the trend until 2020. In the case of CRED origin, the trend has been maintained over the 11 years with values above 90% for children under five years old who enroll in the CRED program in government facilities. The locations where children under five years old have had access to CRED since 2010 have been predominantly in primary care centres with ∼51% and ∼10% in hospitals. However, 30 to 34% of children did not have access to the CRED program since 2010. By 2020, the percentage of CRED attentions in primary care centres dropped to 37%, and of children without CRED grew to 53%. In the case of the maternal education, the predominant educational level reached in the 11 years was secondary education. Since 2015, an increase in the percentage of women with superior education has been observed, maintaining this trend until 2020 with levels above 30%. In the wealth index, the trend has continued over the 11 years, with ∼70% of mothers being concentrated in quintiles 1, 2, and 3. The complete proportions are shown in Table 1.

**Table 1:** Evolution of sociodemographic characteristics during 2010–2020.

		2010	2011	2012	2013	2014	2015	2016	2017	2018	2019	2020
		% (95% CI)	% (95% CI)	% (95% CI)	% (95% CI)	% (95% CI)	% (95% CI)	% (95% CI)	% (95% CI)	% (95% CI)	% (95% CI)	% (95% CI)
**Residence type**												
	**Rural**	36 (34–37)	35 (33–36)	34 (33–36)	32 (30–33)	30 (28–31)	27 (25–28)	27 (26–28)	26 (25–28)	26 (25–27)	26 (25–27)	24 (23–25)
	**Urban**	64 (63–66)	65 (64–67)	66 (64–67)	68 (67–70)	70 (69–72)	73 (72–75)	73 (72–74)	74 (72–75)	74 (72–75)	74 (73–75)	76 (75–77)
**CRED (origin)**												
	**Public**	92 (90–93)	92 (91–93)	94 (93–95)	92 (90–93)	92 (91–93)	91 (90–92)	90 (89–92)	92 (91–93)	90 (89–91)	91 (90–92)	91 (90–93)
	**Private**	8 (7–10)	8 (7–10)	6 (5–7)	8 (7–10)	8 (7–9)	9 (8–10)	10 (8–11)	8 (7–9)	10 (9–11)	9 (8–10)	9 (7–10)
**CRED (facilities)**												
	**Primary health center**	51 (49–53)	52 (50–53)	52 (51–54)	51 (49–53)	52 (50–54)	51 (50–52)	53 (51–54)	51 (49–52)	51 (50–52)	53 (51–54)	37 (35–38)
	**Hospitals**	9 (8–10)	10 (9–11)	10 (9–12)	10 (9–11)	10 (9–12)	12 (11–13)	11 (10–11)	11 (10–12)	12 (11–12)	11 (11–12)	6 (5–7)
	**Private**	5 (5–6)	5 (4–6)	4 (3–5)	6 (5–7)	6 (5–6)	6 (5–7)	7 (6–8)	6 (5–6)	7 (6–8)	6 (6–7)	4 (3–5)
	**No CRED**	34 (33–36)	33 (31–34)	33 (31–35)	33 (32–35)	32 (31–34)	31 (60–32)	30 (29–31)	32 (31–33)	30 (29–31)	30 (29–31)	53 (51–54)
**Maternal education**												
	**No education**	3 (2–3)	3 (3–4)	3 (2–4)	3 (2–3)	2 (2–2)	2 (1–2)	2 (1–2)	1 (1–2)	2 (1–2)	2 (1–2)	1.5 (1–2)
	**Primary school**	33 (31–34)	30 (28–32)	28 (27–30)	26 (25–28)	24 (22–25)	23 (22–24)	22 (21–23)	20 (18–21)	19 (18–20)	18 (17–19)	16.5 (16–17)
	**Secondary school**	44 (42–46)	44 (42–46)	45 (44–47)	46 (44–48)	48 (46–49)	46 (45–48)	46 (45–47)	47 (45–48)	44 (43–45)	46 (45–47)	47 (46–49)
	**Superior education**	21 (19–23)	22 (21–24)	23 (22–25)	25 (23–26)	27 (25–28)	29 (28–30)	30 (29–31)	32 (31–34)	35 (34–36)	35 (34–36)	35 (34–36)
**Wealth index**												
	**1st**	25 (23–27)	25 (23–26)	24 (22–25)	25 (23–27)	24 (22–25)	23 (22–24)	22 (21–24)	24 (22–25)	25 (24–26)	25 (24–26)	23 (22–24)
	**2nd**	23 (21–25)	23 (21–24)	24 (22–26)	23 (22–25)	24 (22–26)	22 (21–23)	23 (22–25)	24 (23–25)	23 (22–24)	24 (23–25)	24 (23–25)
	**3rd**	22 (20–23)	22 (20–24)	22 (21–24)	22 (20–23)	21 (20–23)	20 (19–21)	21 (20–22)	20 (19–21)	20 (19–21)	20 (19–20)	21 (20–22)
	**4th**	17 (16–19)	17 (16–19)	17 (16–19)	17 (16–19)	18 (16–19)	18 (17–19)	18 (17–19)	18 (17–19)	17 (16–18)	17 (16–18)	17 (16–18)
	**5th**	13 (11–14)	13 (11–15)	13 (11–15)	13 (11–15)	13 (12–15)	16 (15–18)	16 (14–17)	14 (13–15)	15 (14–16)	14 (14–15)	15 (14–16)

95% CI: 95% Confidence Interval.

**Figure 1:**
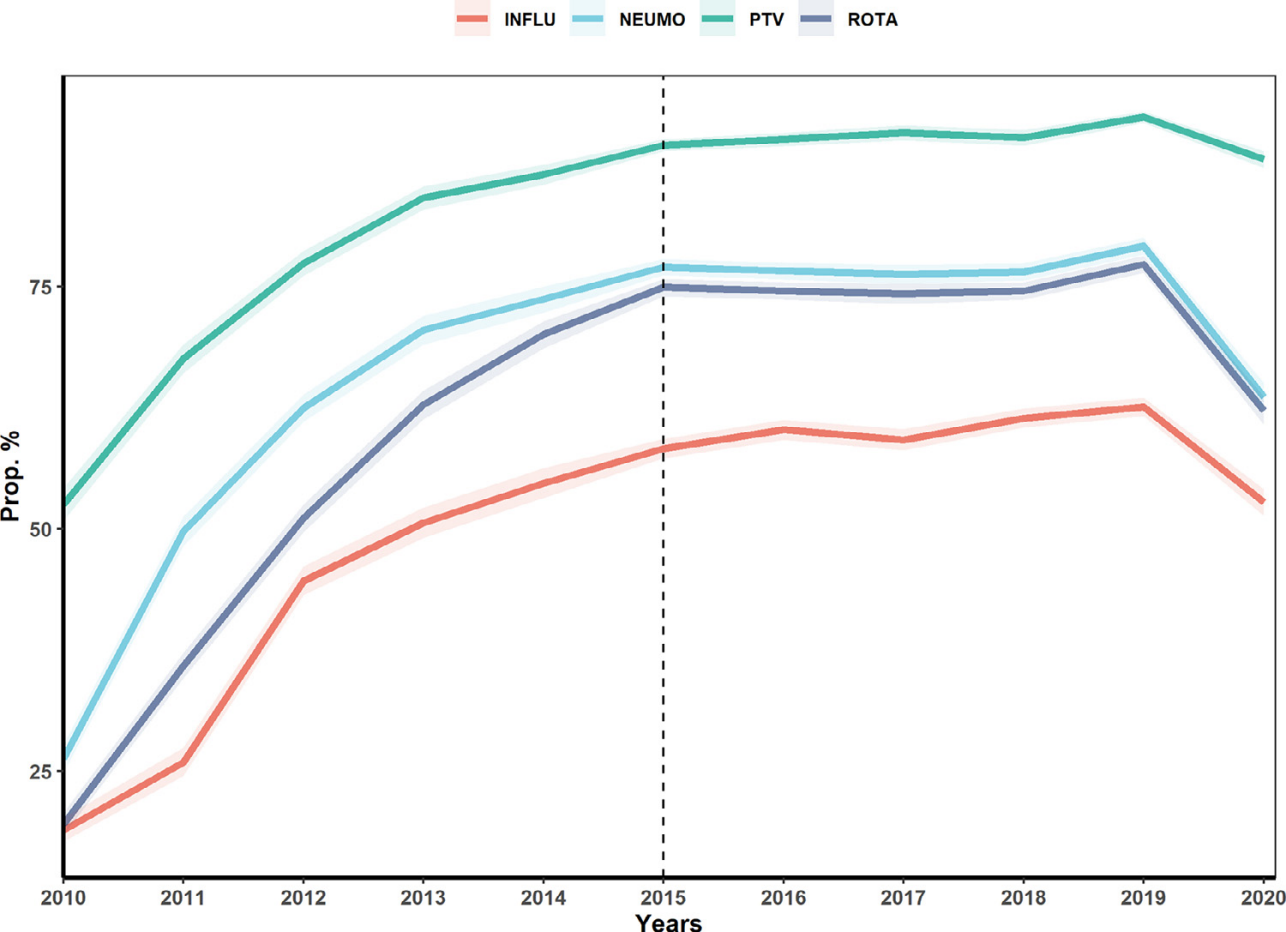
**Vaccination coverage 2010-2020.** Coverage of the first doses of pentavalent (PTV), Pneumococcal (NEUMO), Influenza (INLFU and Rotavirus vaccine (ROTA). The dashed line indicates the start of the new sampling methodology.

**Figure 2:**
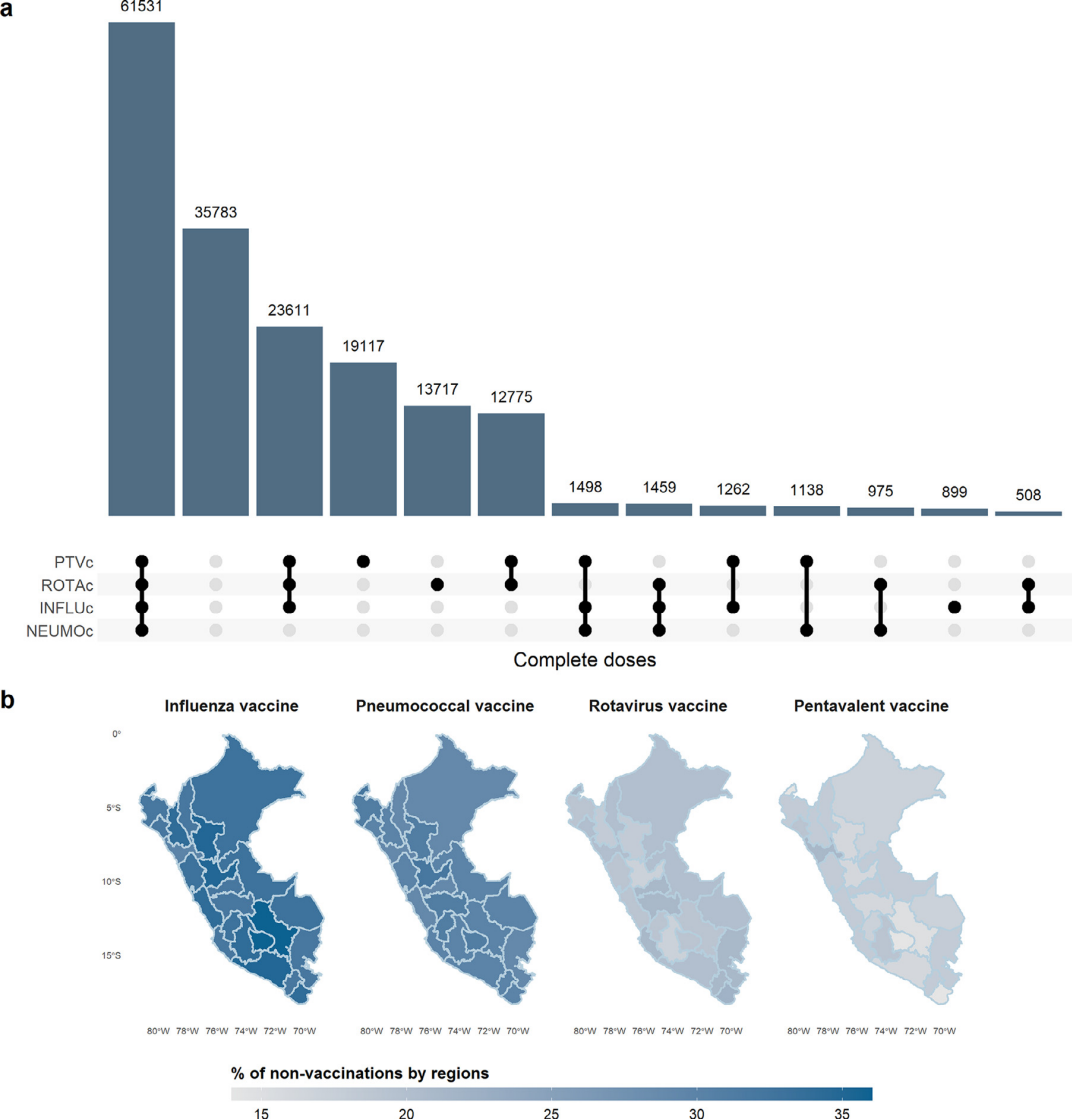
**Distribution of complete doses of vaccine. a.** Doses completes of four vaccines for under five years calculated with the unweighted population of the 11 years. **b.** Complete vaccination schedule for each vaccine by department.

Vaccination coverage (1st dose) over the 11 years has shown important changes. In the case of the pentavalent vaccine (PTV), vaccination coverage in 2010 was 52·5% (CI 95% 50·9–54·2), reaching the expected values only in 2014 with 86.6% (CI 95% 85·5–87·6), increasing coverage until 2019 with 92·5% (CI 95% 91·9–93·1) and then dropping to 88·1% (CI 95% 87·3–89) in 2020. In the case of pneumococcal vaccine (NEUMO), vaccination coverage was 26·3% (CI 95% 24·9–27·7) in 2010, reaching its maximum value in 2019 with 79·2% (CI 95% 78·3–80) and dropping to 63·6% (CI 95% 62·1–65) in 2020. Influenza vaccine (INFLU) had a coverage of 19% (CI 95% 17·8–20·2) for 2010, increasing progressively until 2019 with 62·6% (CI 95% 61·6–63·5) and dropping to 52·7% (CI 95% 51·3–54·1) in 2020. And lastly, rotavirus vaccine (ROTA), started in 2010 with 19·5% (CI 95% 18·4–20·7) coverage, increasing to 77·3% (CI 95% 76·4–78·2) in 2019 and then dropping to 62·2% (CI 95% 60·7–63·7) in 2020 (Figure 1). Figure 2A. shows the unweighted distribution of children under five years with complete vaccination schedules for each vaccine and their combinations for the 11-year period. Note that the second most frequent case is not having any complete vaccination schedule. Figure 2B. shows the percentage of non-vaccination by department, with the influenza and pneumococcal vaccines having the highest levels of non-complete vaccination at the departmental level. The evolution of vaccination coverage by doses for each department for the 11 years is shown in Supplementary Figure 1.

**Figure 3:**
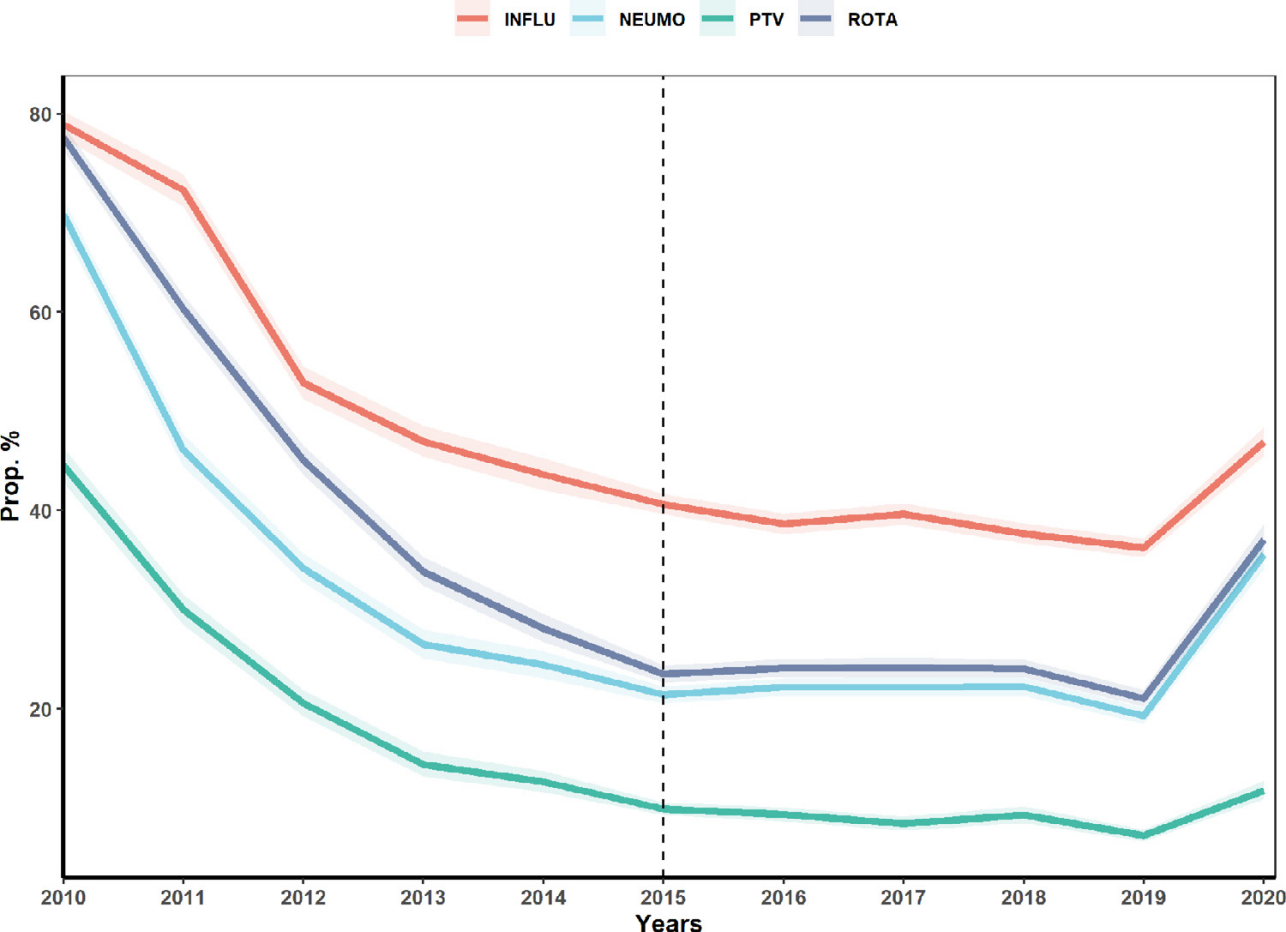
**Evolution of MOVs for each vaccine during the 11-years period**. MOVs of pentavalent (PTV), Pneumococcal (NM), Influenza (INLFU) and Rotavirus vaccine (ROTA). The dashed line indicates the start of the new sampling methodology.

Similar to vaccination coverage, MOVs have noticeably changed over the 11-year period. The four vaccines, by 2010, had very high levels of MOVs at the national level, with 44·5% (CI 95% 42·8–46·3), 69·8% (CI 95% 68·2–71·3), 78·9% (CI 95% 77·5–80·2) and 77·6% (CI 95% 76·3–78·8) for PTV, NEUMO, INFLU, and ROTA vaccines, respectively. In the case of the PTV vaccine, it quickly reached 9·9% (CI 95% 9·3–10·6) for the year 2015 in contrast to the other vaccines, reaching its lowest point in 2019 with only 7·2% (CI 95% 6·6–7·8). NEUMO vaccine reached its lowest level in 2019 at 19·3% (CI 95% 18·5–20·1), and INFLU vaccine was the one that decreased its MOVs more slowly, only reaching its lowest value in 2019 with 36·2% (CI 95% 35·3–37·2). ROTA vaccine, despite it being the vaccine with the second highest MOVs, had the most drastic change in 2019, with 21·1% (CI 95% 20·2–21·9). By 2020, MOVs in all four vaccines raised again, with 11·8% (CI 95% 10·9–12·7), 35·6% (CI 95% 34·1–37·1), 46·9% (CI 95% 45·4–48·5), and 37·1% (CI 95% 35·5–38·8) for PTV, NEUMO, INFLU, and ROTA vaccines, respectively (Figure 3).

### Estimation of SII for MOVs and spatial analysis

**Figure 4:**
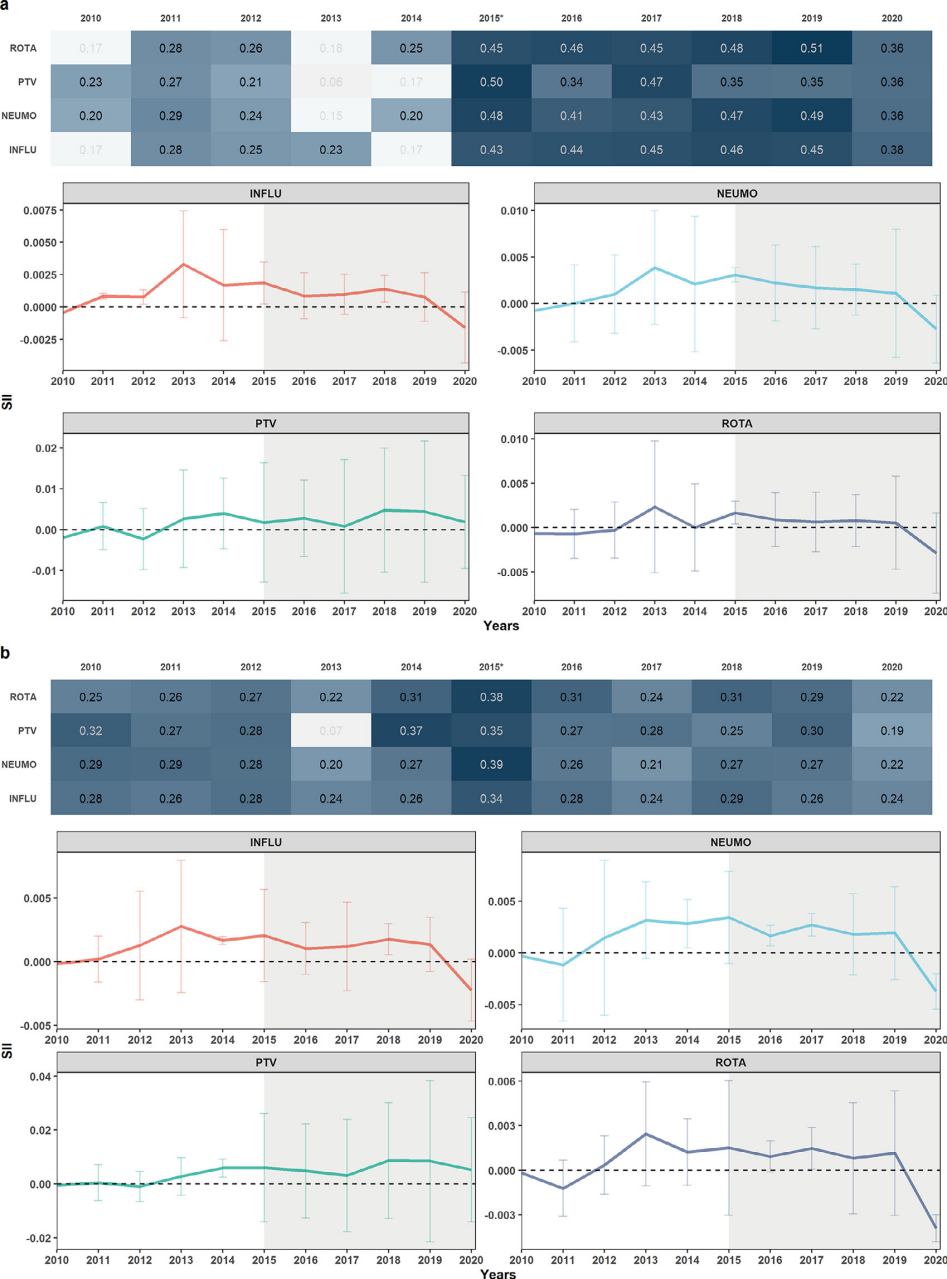
**Slope Index of Inequality at national level for the four vaccines. a.** Maternal Education. **b.** Wealth index. Evolution of SII of the four vaccines during the 11 years at national level and *Moran's I* test results. A heatmap is shown with the results per year and per vaccine with *Moran's I* test estimates (top). Estimates with non-significant p-values are transparent. The evolution of SII for each vaccine during the 11 years is shown with its CI (bottom). The shaded area indicates the start of the new sampling methodology.

At the national level, drastic changes over time were observed for WI and ME inequalities. In the case of WI, all four vaccines in 2010 had negative SII (MOV rate is higher in the lowest category). By 2013, this SII changed to positive for all four vaccines until 2020 when they became negative again except for the PTV vaccine. In the spatial analysis, a significant spatial correlation was observed every year, during the 11 years, with estimates between 0·19 and 0·39 for the *Moran's I* test. By 2015, all four vaccines had the highest level of spatial autocorrelation, decreasing progressively until 2020**.** In the case of ME, all four vaccines in 2010 had a negative SII value. In 2011, PTV, NEUMO, and INFLU became SII positive, except ROTA, which remained negative until 2012. By the same year, PTV became negative again but, from 2013, all vaccines maintained a positive SII until 2019. In 2020, all returned to negative SII, except PTV. In the spatial analysis, significant spatial correlation was observed in almost every year. In the first five years, an estimated *Moran's I* test of up to 0·51 was observed for all four vaccines, values of almost double of what was obtained between 2010 and 2014. Figure 4, shows the *Moran's I* test estimates and the point and interval estimates of SII for the 11 years for each vaccine for Wealth index and Maternal education (4a and 4b, respectively). It is important to take into account the change in the sampling methodology at this point of the analysis. In Figure 4. we observe that, starting in 2015, a considerable increase in the correlation coefficient for the 4 vaccines is evident for both socioeconomic variables (almost twice as high for Maternal Education). This increase is maintained until 2019. In the case of MOVs and vaccination coverage trends, no drastic changes are observed until 2019.

**Figure 5:**
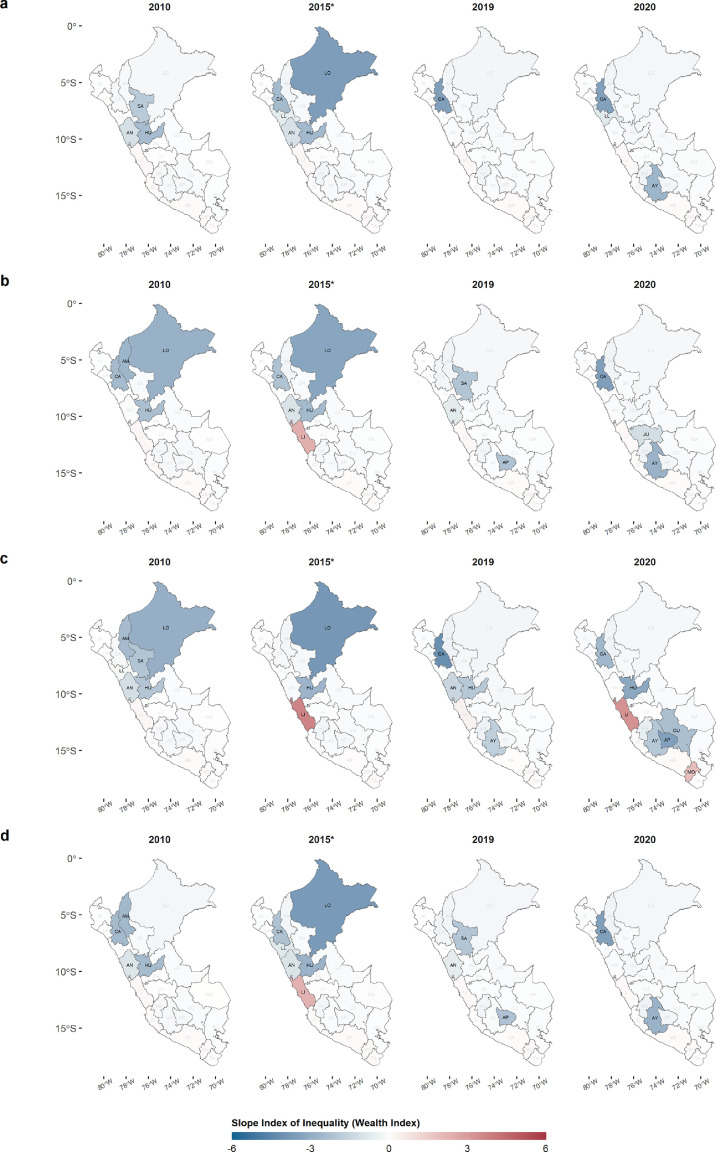
**Slope Index of Inequality of the MOVs for each department from 2010 to 2020 (Wealth index, WI).** The 4 years are shown as a reference of SII variations during the 11 years of study. A. INFLU, B. PNEUMO, C. PTV, D. ROTA. Abbreviations of departments in Supplementary Table 2.

**Figure 6:**
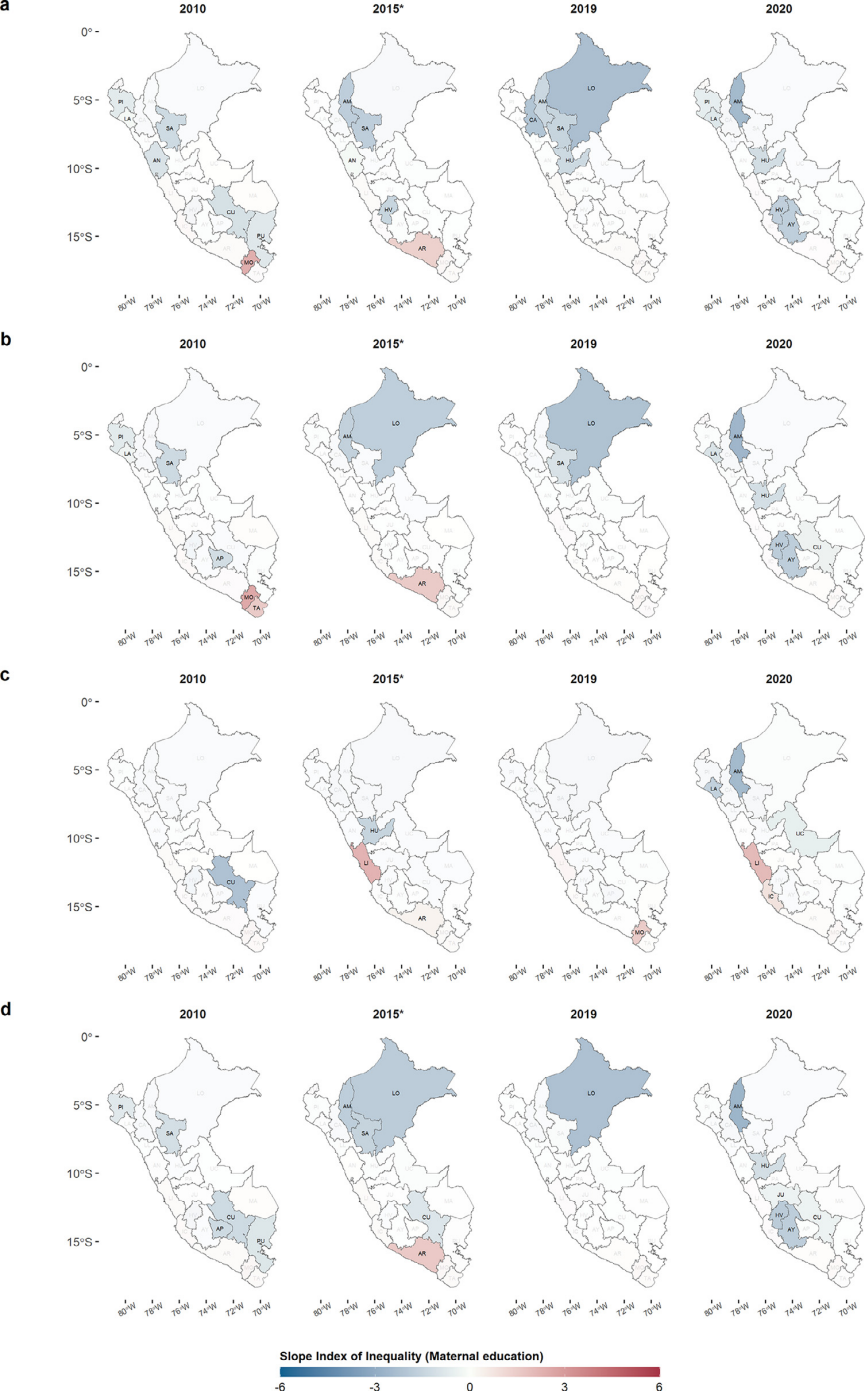
**Slope Index of Inequality of the MOVs for each department from 2010 to 2020 (Maternal Education, ME).** The 4 years are shown as a reference of SII variations during the 11 years of study. A. INFLU, B. PNEUMO, C. PTV, D. ROTA. Abbreviations of departments in Supplementary Table 2.

At the departmental level, the SIIs for each socioeconomic variable were positive only in five coastal departments (Lima, Ica, Arequipa, Moquegua, and Tacna). Figure 5 shows four years of the total study period for inequality in WI, where it is observed that the departments of the central Andes and the north of the country as well as Loreto (the largest Amazon department), had negative SII values with significant values for each vaccine. All vaccines except INFLU had the Lima department with a positive SII, with a significant value, with PTV ending the decade with the same pattern. In the case of department maternal education inequality, Figure 6 shows the same pattern in the central and northern Andes departments, as well as in the Amazon departments, such as Loreto and Ucayali, with negative SII values. In addition, coastal departments such as Lima, Arequipa, Moquegua, and Tacna have positive SII with significant values throughout the decade, with PTV being the only one that ended the decade with a positive SII value in Lima. The complete department evolution of SII for the four vaccines is presented in Supplementary Figures 2 and 3.

### Stratified SII analysis

**Figure 7:**
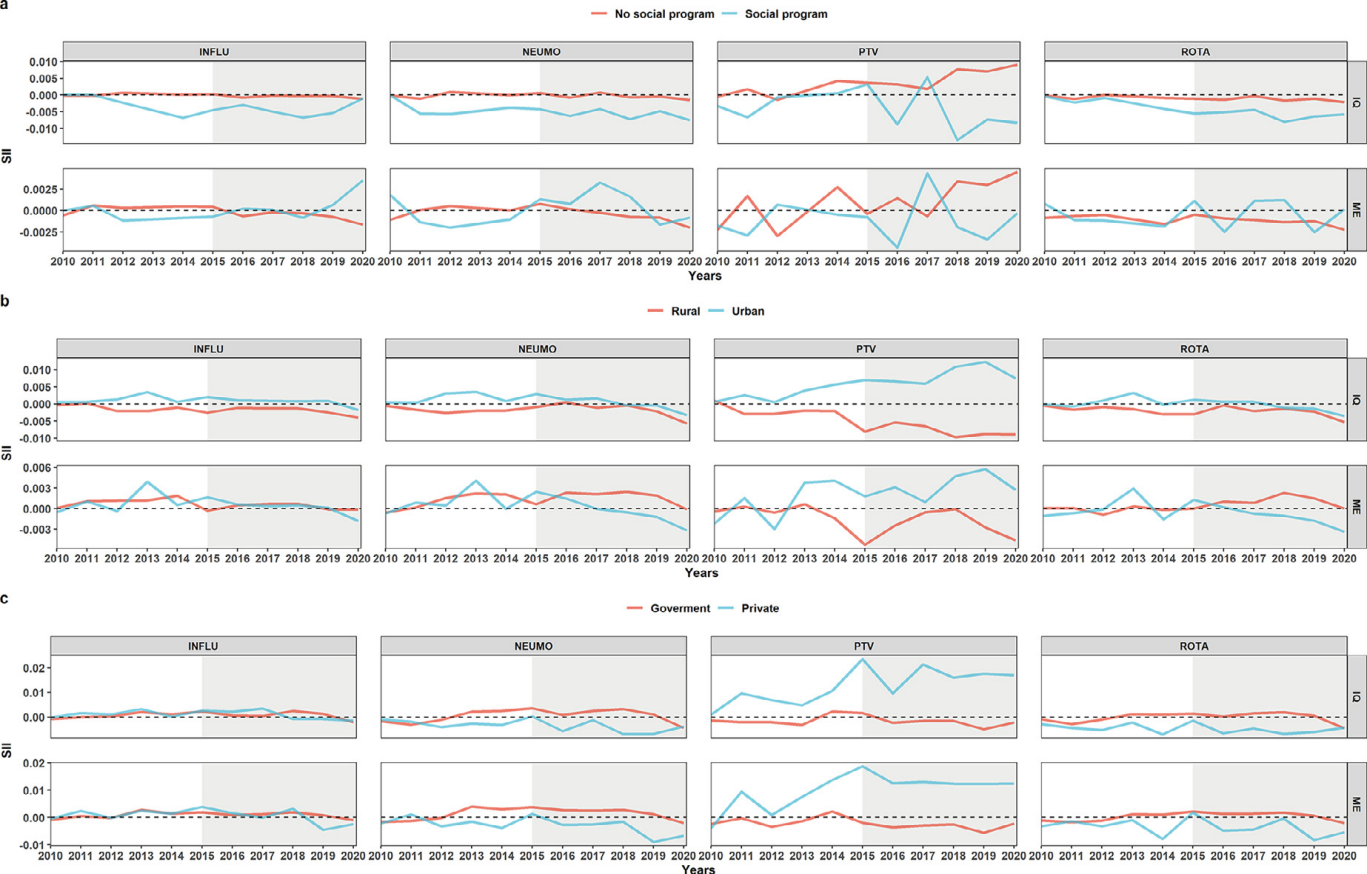
**Stratification of the Slope Index of Inequality analysis. and B**. Stratification by access and non-access to the JUNTOS program, respectively. **C and D**. Stratification by housing in rural and urban areas, respectively. **E and F.** Stratification by CRED care in a government and private facility, respectively. **WI:** Wealth index. **ME:** Maternal Education. The shaded area indicates the start of the new sampling methodology.

The stratification of the SII analysis at the national level by access to the JUNTOS program, showed that those who had access to this program concentrated greater WI inequality in the lowest category (negative SII), and this trend was maintained during the 11 years, except for the PTV vaccine, which did not have a marked trend. However, there were no marked differences in ME inequality between those who were or were not in a social program. In the case of stratification by residence type, differences were only observed in almost all years between the SII of residence types for PTV. In the case of PNEUMO and ROTA vaccines, a change in SII from positive to negative was observed from 2015 for those living in urban housing. Finally, in the stratification by type of health facility, no differences were observed between groups for the INFLU vaccine. The NEUMO and ROTA vaccines had positive SII values for those attended in government facilities and, contrary to the others, PTV had positive SII values for those attended in private facilities (Figure 7).

## Discussion

The elimination of MOVs is still a pending objective for the Immunization Agenda 2030 mainly in developing countries. In this study, 2010–2020 DHS data were analysed in Peru to show the trends of SII at national and departmental level of MOVs, for the four vaccines that prevent the main causes of death in children < five years, using two socioeconomic variables. We observed a high frequency of MOVs during the 11 years, with its lowest level by 2019, but increasing again by 2020. In addition, by calculating the SII of MOVs for two socioeconomic variables (Maternal education and Wealth index), we found that, at the national level, greater inequality was concentrated in the richest categories of each socioeconomic variable. At the departmental-level analysis, we found that in contrast to the national analysis, only five of the 24 departments had positive SII values during the 11 years. Finally, we observed the presence of clusters of departments whose SII values were correlated and maintained throughout 2010–2020.

In this study, a very high level of MOVs for the four vaccines was evident from the beginning of the decade at the national level, however, these were decreasing during the following years until the lowest point in 2019. This could be explained by the fact that pneumococcal and rotavirus vaccines were included in the national vaccination schedule since 2010.^25^ This change in policy may have had a positive impact, allowing vaccination to become more widespread, and MOVs to decrease as a consequence. Another important finding is the differences of MOVs between vaccines that according to the national immunization schedule, should be given at the same time, such as PTV, ROTA, and INFLU. We found that there are different levels of MOVs for each vaccine during the 11 years. This could be due to the availability of the vaccines due to logistical problems, the lack of information for parents that, as a consequence, refuse multiple applications of vaccines for their children, and the lack of information of health workers, who for fear of the side effects decided to prioritize some vaccines over others. These differences are important because they show that vaccine refusal could be linked to specific fears, that could be addressed with appropriate information. They also show that some people could reject one vaccine, but that does not mean that they would reject all vaccines. Similar immunization behaviors were observed in the other countries of the South American region, where, by 2010, only 61% of the countries reached the pentavalent vaccine (PTV) immunization coverage targets proposed by the GVAP, and by 2019, only 36%.^26^ The same occurs for other vaccines such as anti-hepatitis B and rotavirus. Individually, in the last decade, the countries that have seen a decrease in immunization coverage with the pentavalent vaccine in the region are Brazil, Bolivia, Argentina, Uruguay, Paraguay, and other Caribbean countries.^27^

The values obtained for MOVs in this study are similar to those reported in developing countries,^27^, ^28^, ^29^ possibly because they share health systems that cannot close the gaps that exist within countries, enhanced by existing socioeconomic and health inequalities. This idea becomes stronger if we take into account that, despite currently having a very low frequency of MOV at the national level for the pentavalent vaccine, in 2021 there was an outbreak of diphtheria in Peru after 20 years of eradication.^30^^,^^31^ The relationship between these socioeconomic inequalities and health has been extensively described.^32^, ^33^, ^34^, ^35^ In relation to this, we identified that, during the 11 years, almost one third of the children under five years old were not included in the growth and development control program given by the government, possibly because there are still areas where children are not born in a health centre or the distance to these is excessively far (geographic accessibility).^36^ Moreover, not being in a monitoring program would mean a higher risk of the occurrence of cases of immuno-preventable infections. On the other hand, social (maternal education, social programs, governance) and economic (wealth index) characteristics significantly affect the increase in MOVs and low vaccination coverage.^33^ In this study, using the SII, we obtained that, at the national level, the greatest inequality of MOVs was concentrated at the richest level, for both maternal education and the wealth index, except for 2020, when the SII became negative. These results are different from those reported in Senegal, Bangladesh, Bolivia and Guinea,^33^ which obtained higher inequality in the poorest strata, calculating the SII considering vaccination coverage as the health event. These differences could be due to the fact that these countries have higher national poverty rates (∼40%) compared to Peru, which registers a poverty level of 20% for 2020.^37^

Although at the national level there was greater inequality in the higher categories for both wealth index and maternal education, at the departmental level we observed that there were departments with contrasting SII values. The Lima department remained with positive values for SII of MOVs in both socioeconomic variables during the 11 years, in contrast from what was found in the northern and central zone of the Andes, where the greatest inequality was concentrated during the 11 years in the lowest levels of income and education. This is possibly due to the fact that the departments with negative SII are, at the same time, the poorest departments of Peru, with 47%, 46% and 44% of poverty for the Huancavelica, Ayacucho, and Pasco departments, respectively for 2020.^38^ Poverty negatively influences the performance of health systems, which translates into deficiencies in both health promotion and prevention.^37^^,^^39^ Besides, JUNTOS program, that targets poor people focusing mainly on the poorest regions and rural areas, has as one of the conditionalities, the vaccinations of under five years old. JUNTOS has contributed to improving vaccination coverage among the poorest. However, the level of MOVs among the wealthier show the risk of prioritizing health policies based on economic conditionalities without improving the capacity of the health system to secure access to health services and information, as well as the availability of supplies such as vaccines. Our results show the positive values for SII in departments such as lima, Ica, Arequipa, and Moquegua, which have the lowest percentage of poverty (∼20%), which have had better health systems over the years. In these regions, population, may have the perception that vaccines do not have a major impact on children's health or, in the worst case, be influenced by alarmist groups such as anti-vaccine groups.^19^^,^^40^ Moreover, we found that these departments with similar SII values formed clusters of departments with similar SII behaviors over the years, located in the central Andes, South and Amazon departments. This possible explanation for the heterogeneity observed within the country would be reinforced by the evolution of vaccination against COVID-19 in the last year, as departments with lower poverty rates and better health systems had a higher vaccination rate compared to poorer departments.^41^

The COVID-19 pandemic had a significant impact on the health system of developing countries like Peru. The pandemic required institutions to redirect funding, health professionals and logistics to detect and attend COVID-19 cases since the beginning of 2020, which caused the neglect of other non-COVID and chronic diseases, growth and development control in children under five years old, and immunizations.^42^ At the regional level, an average vaccination coverage of 80% for the pentavalent vaccine in South America was expected for 2020, but by the end of the year only 75% was achieved.^43^ As of March 2020, vaccination coverage for pentavalent vaccine dropped to almost 60%, remaining below expectations until September and October, when it began to rise again.^43^ This study shows an increase in MOVs at the national and departmental level by 2020, however, in the departments most affected in terms of number of cases and deaths due to COVID-19, such as Loreto, Madre de Dios, Puno, and La Libertad, there was a greater decrease in vaccination coverage by 2020.^44^

This study has a number of limitations. A secondary data source was used that is aimed to collect information about population health, but it is not designed for a comprehensive study of MOVs. Using the WHO case definition of MOVs and the available survey data, we constructed the MOV variable that allowed us to perform the analysis. Another limitation is the type of sampling used for the surveys. For surveys before 2015, it was detected that the sampling type used generated a high variability in the basic indicators of the survey. Due to this reason, since 2015, the type of sampling was changed to a balanced sampling (cube sampling). Considering this, we suggest taking into account this difference in sampling, and also consider the variability declared for the data reported before 2015. Finally, there are variables that were added to the DHS in recent years (e.g., social programs), so only the years for which complete data were available were considered for the analysis.

In conclusion, there was a high heterogeneity of MOVs and a high concentration of MOVs in the lower levels of socioeconomic variables at the national and departmental level. This evidence highlights the inequity at the national and departmental levels for more than a decade, which would help policy makers to focus on the departments with the highest priority to improve vaccination programs, taking into account the multidimensionality of the causes of missed opportunities for vaccination.

## Contributors

JM-C and GC-E conceived the idea of the study, design of the study, and analyzed the data. JM-C, GC-E, and CG wrote the manuscript. All authors reviewed and approved the final manuscript.

## Data sharing statement

The data and code used to produce the analysis is open access and freely available from https://github.com/healthinnovation/MOV and archived in a permanent repository.

## Editor note

The Lancet Group takes a neutral position with respect to territorial claims in published maps and institutional affiliations.

## Declaration of interests

The authors declare no competing interests.

## Appendix Supplementary materials

Image, application 1
