# Supplementary material for: Missed opportunities for vaccination in Peru 2010–2020: A study of socioeconomic inequalities
Source: Lancet Reg Health Am. 2022 Jul 18;14:100321. doi: 10.1016/j.lana.2022.100321 (PMC9904149; doi:10.1016/j.lana.2022.100321)
Supplement: Supplementary file 1 [file mmc1.docx]

**Supplementary Figure 1.** Vaccination schedules per dose during the last 11 years for each region. **A.** Pentavalent vaccine doses. **B.** Pneumococcal vaccine doses. **C.** Influenza vaccine doses. **D.** Rotavirus vaccine doses. The dotted lines indicate 85% coverage.


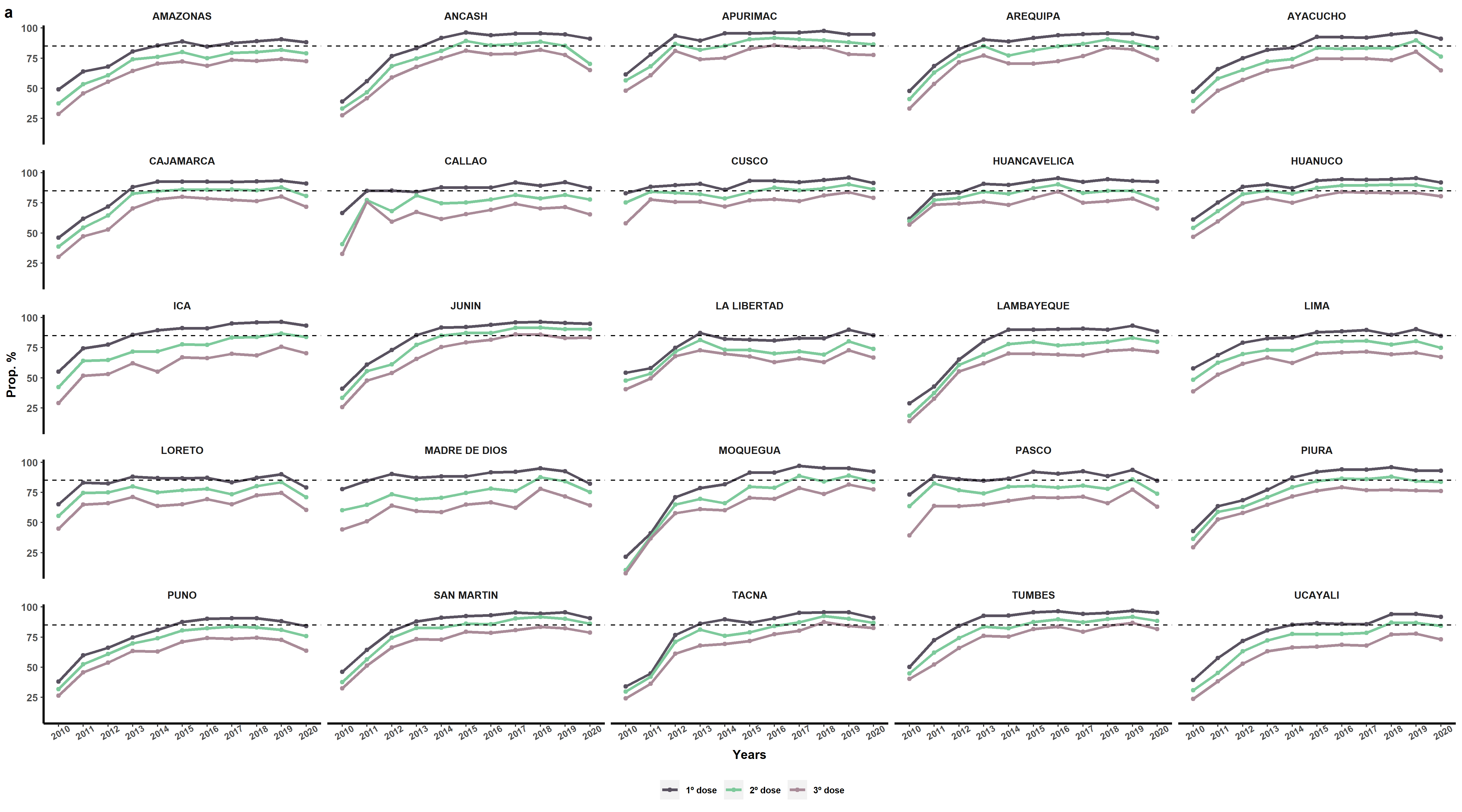


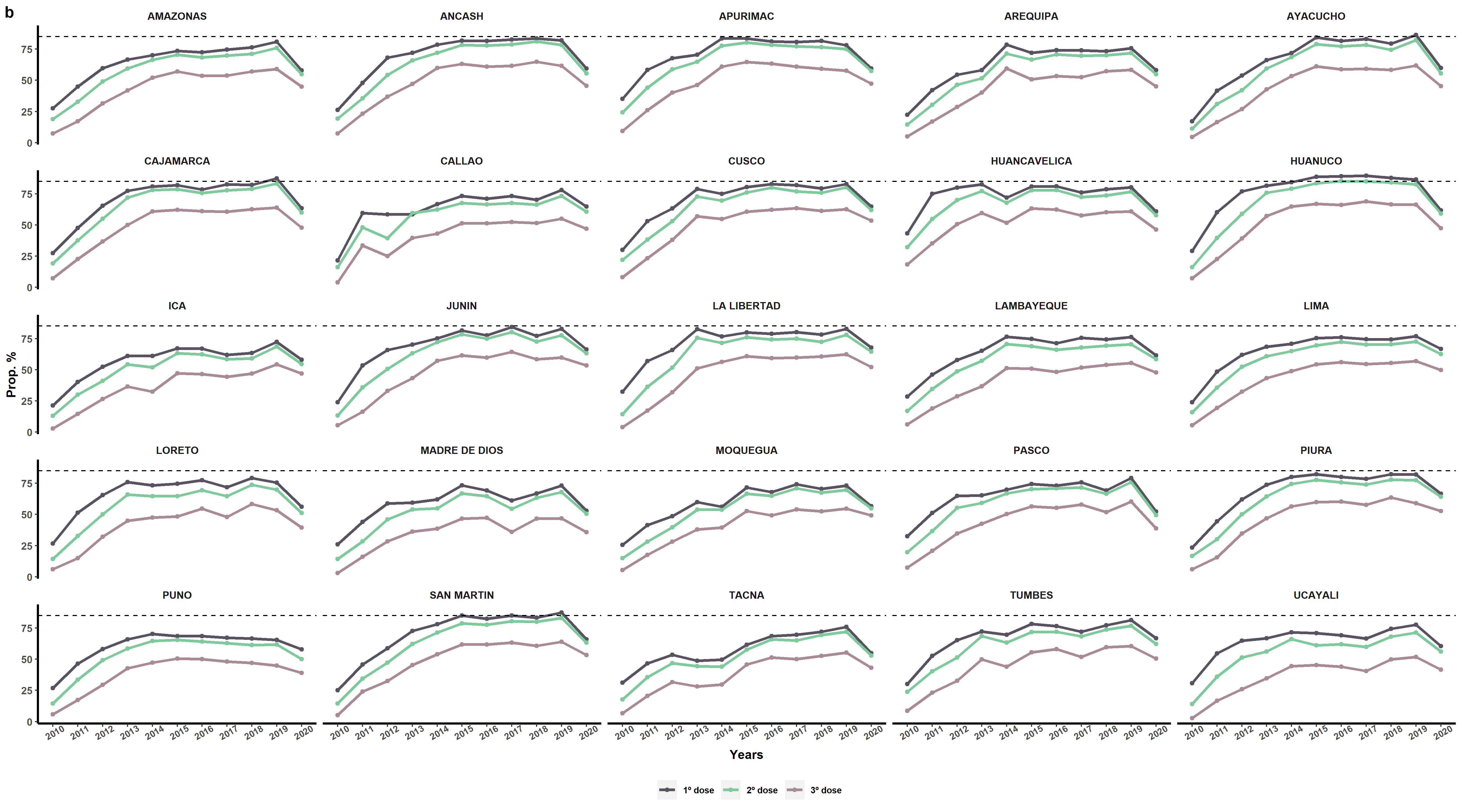


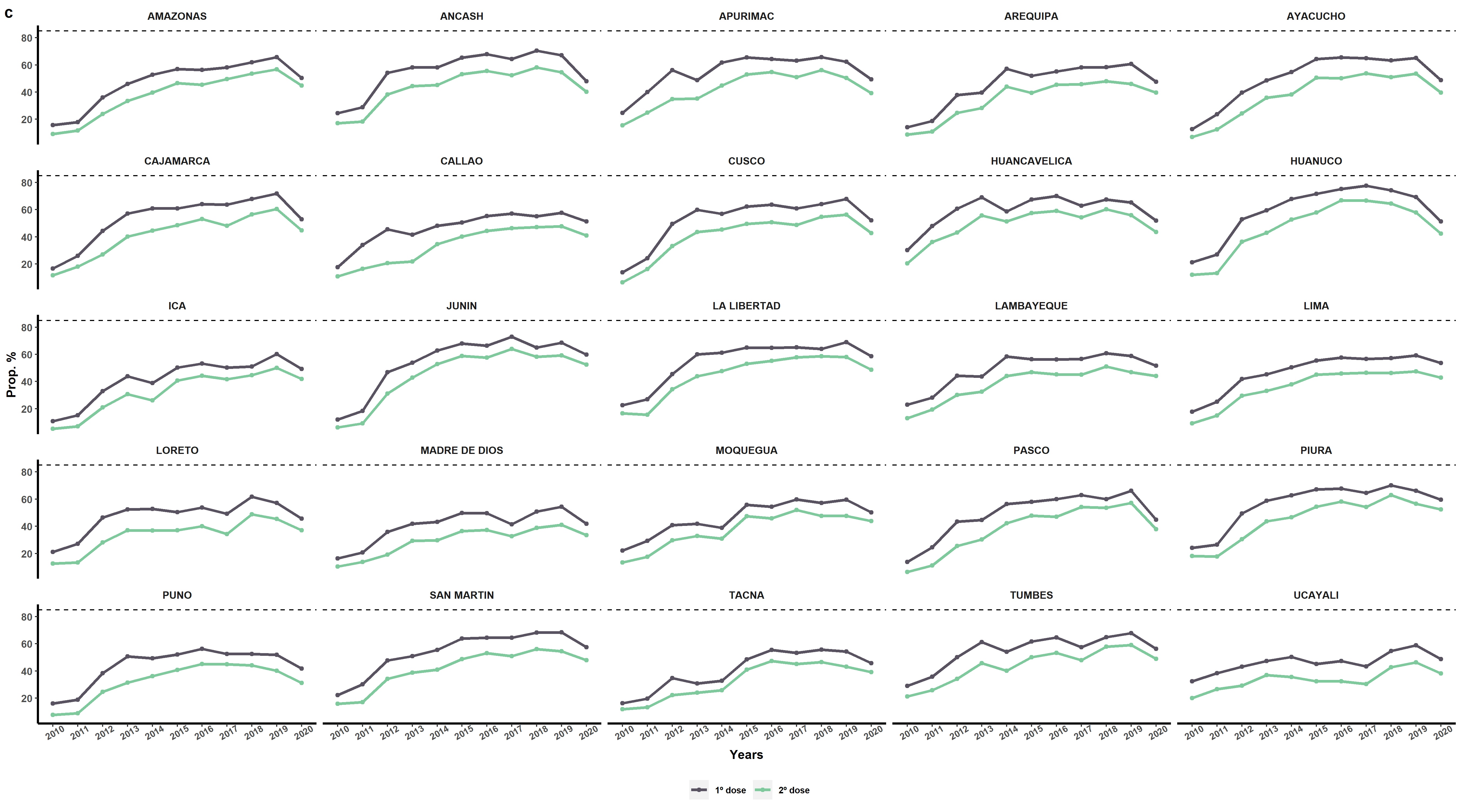


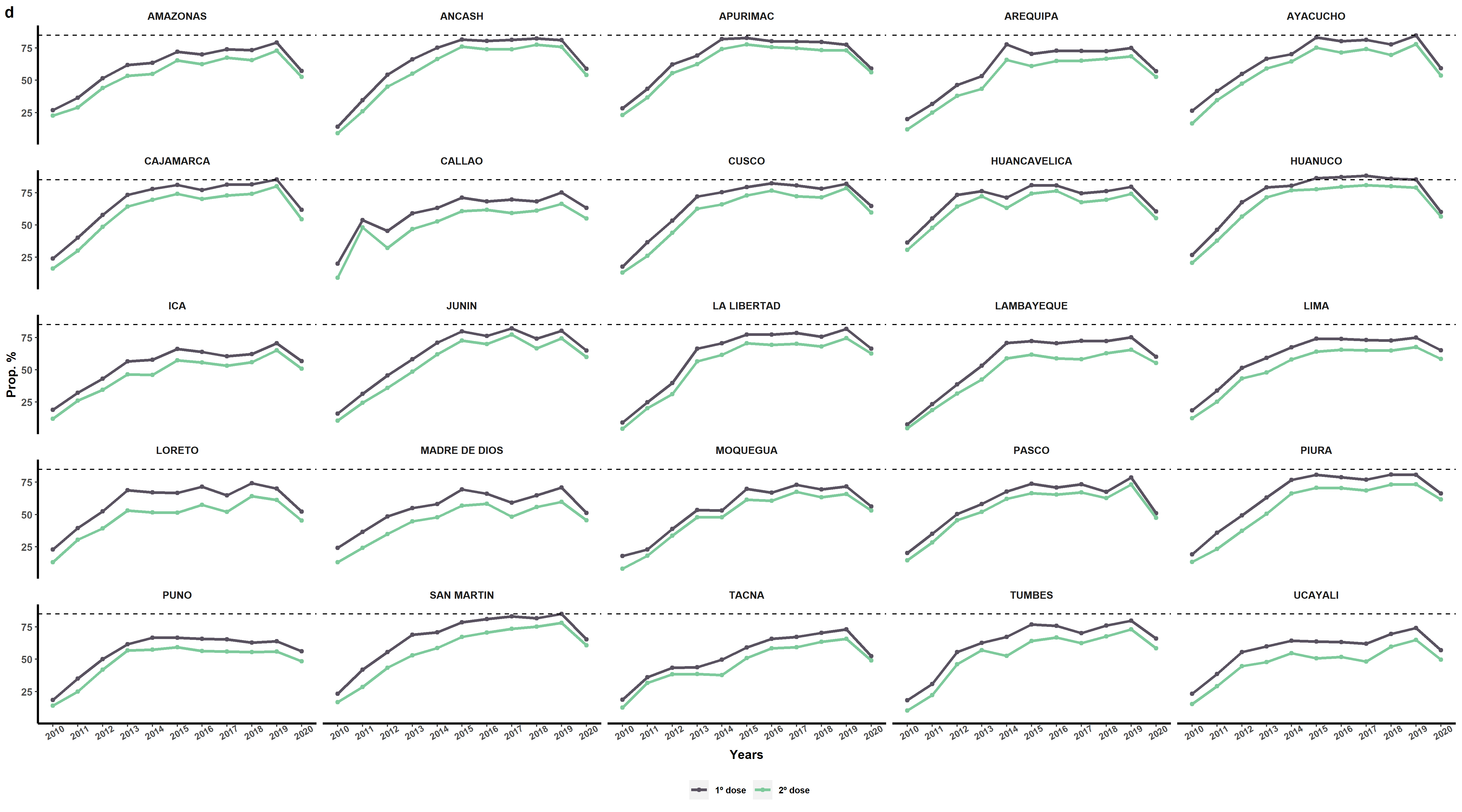


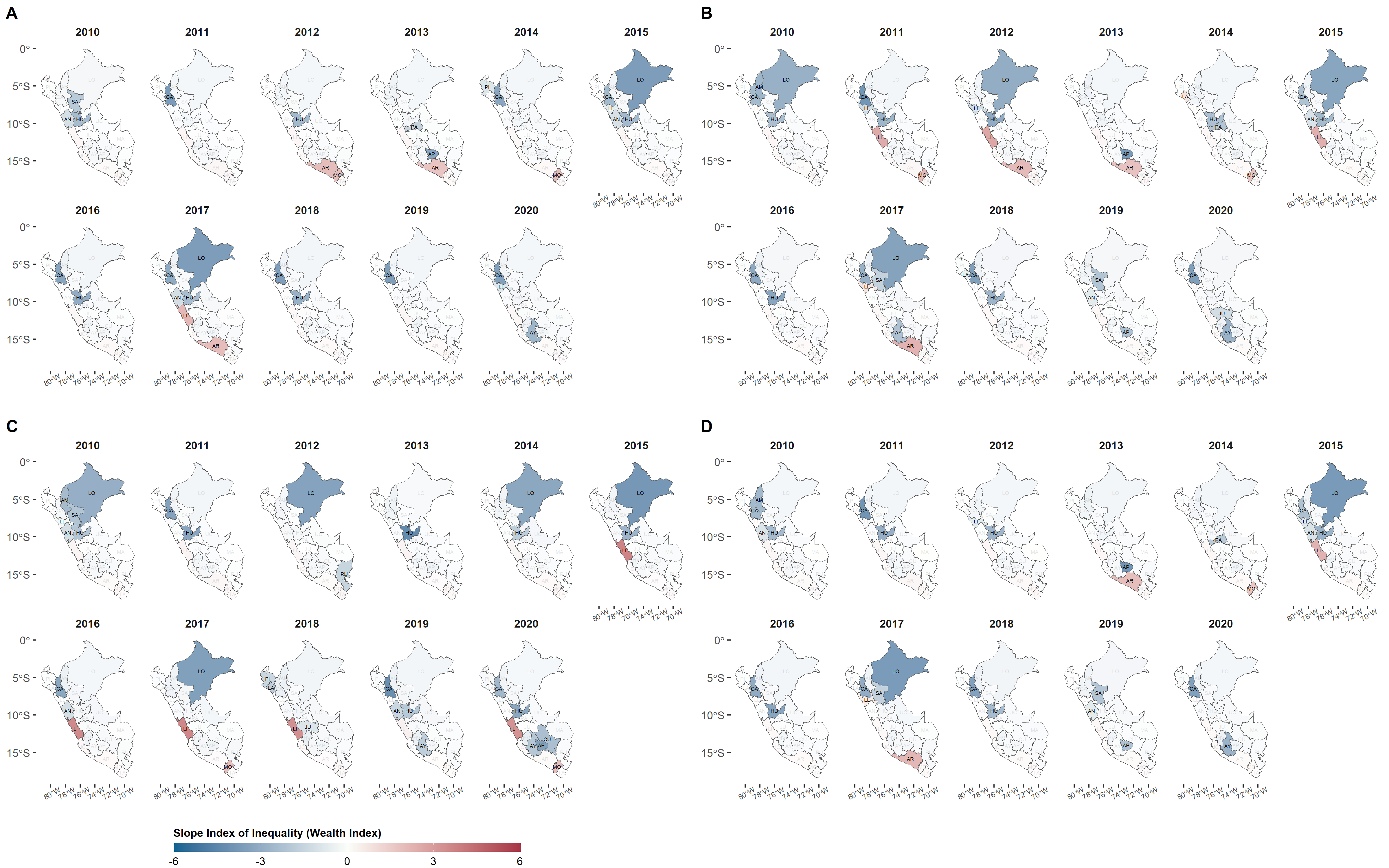
**Supplementary figure 2. SII of the MOVs for each region from 2010 – 2020 (Wealth Index, WI).** The 4 years are shown as a reference of SII variations during the 11 years of study. A. INFLU, B. PNEUMO, C. PTV, D. ROTA.


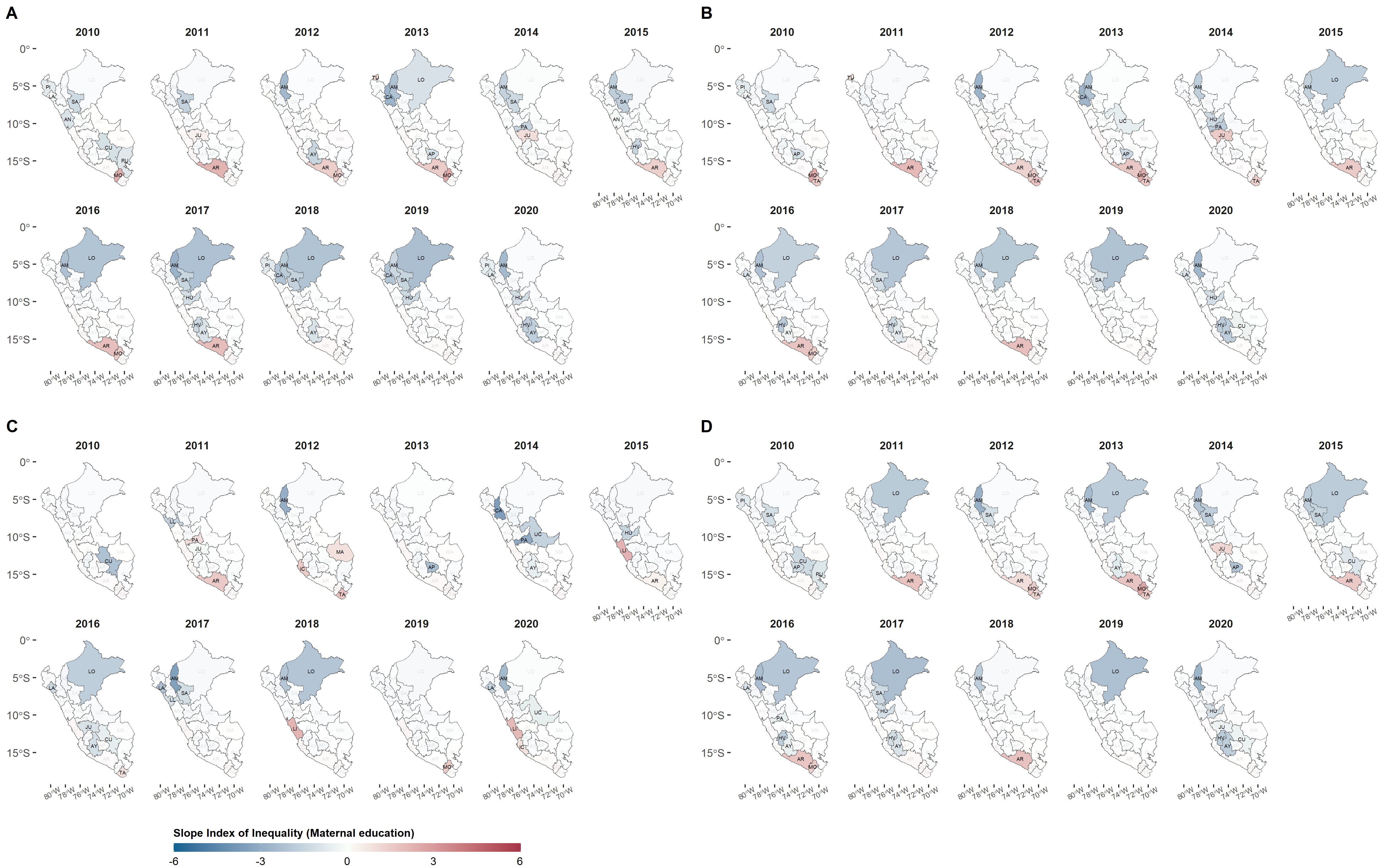
**Supplementary figure 3. Fig. 6. SII of the MOVs for each region from 2010 – 2020 (Maternal Education, ME).** The 4 years are shown as a reference of SII variations during the 11 years of study. A. INFLU, B. PNEUMO, C. PTV, D. ROTA.

| **Supplementary table 1. National vaccination schedule for children under 5 years old** | | |
| --- | --- | --- |
| **Target population** | **Age** | **Vaccine** |
| under one year old | newborn | 1 dose of BCG vaccine |
|  |  | 1 dose of HVB vaccine |
|  | 2 months | 1 dose of pentavalent vaccine |
|  |  | 1 dose of inactivated polio vaccine (parenteral) |
|  |  | 1 dose of rotavirus vaccine |
|  |  | 1 dose of pneumococcal vaccine |
|  | 4 months | 2 doses of pentavalent vaccine |
|  |  | 2 dose of inactivated polio vaccine (parenteral) |
|  |  | 2 dose rotavirus vaccine |
|  |  | 2 dose pneumococcal vaccine |
|  | 6 months | 3 dose of pentavalent vaccine |
|  |  | 1 dose of polio vaccine (oral) |
|  |  | 1 dose of pediatric influenza vaccine |
|  | at 1 month after the 1st dose of influenza vaccination | 2 dose of pediatric influenza vaccine |
| 1 year old | 12 months | 1 dose MMR vaccine |
|  |  | 3 dose of pneumococcal vaccine |
|  |  | 1 dose of chickenpox vaccine |
|  |  | 1 dose of pediatric influenza vaccine |
|  | 15 months | 1 dose of yellow fever vaccine |
|  | 18 months | 1 booster dose of pentavalent vaccine |
|  |  | 1 booster dose of oral antipolio vaccine |
|  |  | 2 dose of MMR vaccine |
| 2-year-old | 2 years 11 months | 1 dose of pediatric influenza vaccine |
|  |  | 1 dose of chickenpox vaccine |
| 3- and 4-year-old | 3 years 11 months - 4 years 11 months | 1 dose of adult influenza |
| 2-, 3- and 4-year-old | until 4 years 11 months | 1 dose yellow fever vaccine |
| 4-year-old | until 4 years 11 months | 2 booster doses of pentavalent vaccine |
|  |  | 2 booster doses of oral antipolio vaccine |

**Supplementary table 2.** Abbreviations of the 24 regions of Peru.

| **REGION** | **ABREVIATIONS** |
| --- | --- |
| Amazonas | AM |
| Ancash | AN |
| Apurimac | AP |
| Arequipa | AR |
| Ayacucho | AY |
| Cajamarca | CA |
| Cusco | CU |
| Huancavelica | HV |
| Huanuco | HU |
| Ica | IC |
| Junin | JU |
| La Libertad | JLL |
| Lambayeque | LA |
| Lima | LI |
| Loreto | LO |
| Madre de Dios | MA |
| Moquegua | MO |
| Pasco | PA |
| Piura | PI |
| Puno | PU |
| San Martin | SA |
| Tacna | TA |
| Tumbes | TU |
| Ucayali | UC |
